# Off-label use of ceftiofur in one-day chicks triggers a short-term increase of ESBL-producing *E*. *coli* in the gut

**DOI:** 10.1371/journal.pone.0203158

**Published:** 2018-09-11

**Authors:** Mauro M. S. Saraiva, Alexandre L. B. Moreira Filho, Oliveiro C. Freitas Neto, Núbia M. V. Silva, Patrícia E. N. Givisiez, Wondwossen A. Gebreyes, Celso J. B. Oliveira

**Affiliations:** 1 Department of Animal Science, Center for Agricultural Sciences, Federal University of Paraiba (CCA/UFPB), Areia, Paraíba, Brazil; 2 National Institute for Research on Antimicrobial Resistance (INPRA), Porto Alegre, Rio Grande do Sul, Brazil; 3 Department of Veterinary Sciences, Center for Agricultural Sciences, Federal University of Paraiba (CCA/UFPB), Areia, Paraíba, Brazil; 4 Department of Preventive Veterinary Medicine, College of Veterinary Medicine, The Ohio State University, Columbus, Ohio, United States of America; 5 Global One Health Initiative (GOHi), The Ohio State University, Columbus, Ohio, United States of America; Ross University School of Veterinary Medicine, SAINT KITTS AND NEVIS

## Abstract

This trial was designed to evaluate the off-label use of ceftiofur with Marek’s vaccine in one-day-old broiler chicks, a prophylactic treatment that has been done in some commercial hatcheries, on the emergence of extended-spectrum beta-lactamase producing *Escherichia coli* (ESBL-*E*. *coli*). A total of 168 chicks (Cobb500^®^) were used in a completely randomized design. Birds were assigned to two treatments (Marek’s vaccine plus saline vs Marek’s vaccine plus ceftiofur) and six repetitions, with 14 animals each. Cloacal swabs were collected from 1 to 14 days post-hatch. The majority (86%; p<0.0001) of the ESBL-producing isolates harboring *bla*_CTX-M_ and *bla*_SHV_ genes originated from animals receiving the antimicrobial. None of the isolates were positive for plasmid-mediated AmpC betalactamase genes (*bla*_ACC_, *bla*_CMY-2_, *bla*_DHA_, *bla*_FOX_, *bla*_MOX_ and *bla*_MIR_). These findings indicate that the off-label use of ceftiofur with Marek’s vaccine is associated with the short-term increase in ESBL-producing *Escherichia coli* in the gut of chicks.

## Introduction

The non-therapeutic use of antimicrobial drugs in farm animals is a common practice for disease control and prevention, or to enhance performance. Previous reports demonstrated the link between the off-label antibiotic use of antimicrobials in animals and the increase in antimicrobial resistance [1,2]. Third-generation cephalosporins are among the highest priority critically important antimicrobials to human health [3] and their indiscriminate use is of great concern to public health.

Ceftiofur is commonly administered to day-one chicks together with Marek’s vaccine in certain commercial hatcheries to prevent disease in broilers [4]. Use of ceftiofur in poultry production has been responsible for the increase of resistant *E*. *coli* and *Salmonella* Heidelberg isolates in Canada [5]. The use of ceftiofur has also been reported to be related to the increase of the *bla*_CTX-M_ gene in *E*. *coli* isolates from swine and cattle fecal samples [6]. The potential spread of resistant isolates of *E*. *coli* or even their encoding plasmids through direct contact or ingestion of contaminated food pose a worrying public health risk. The objective of this study was to experimentally investigate the effects of the administration of ceftiofur together with Marek’s vaccine in one-day-old chicks on the emergence of extended-spectrum beta-lactamases (ESBL) producing *Escherichia coli* (*E*. *coli*) in the gut.

## Materials and methods

The experimental proposal was submitted and approved by the Ethics Committee on Animal Use of the Federal University of Paraiba (CEUA/UFPB). Two hundred fertile eggs were obtained from a commercial hatchery (Guaraves Alimentos Ltda, Guarabira, PB, Brazil), originated from Cobb500 breeders with 44 weeks of age. They were placed in egg incubators with standard temperature (37.7°C) and humidity (60%) conditions and automatic turning at each two hours. After hatch, a total of 168 chicks were weighed individually (mean weight 47.0 ± 0.5g) and used in a completely randomized design with two treatments and six repetitions, with 14 animals per repetition. The chicks were kept into boxes (50 x 50 x 50 cm). Each box was equipped with feeder and drinker and was covered with nylon to avoid contamination between boxes by vectors such as flies. Thermo-hygrometers (Oregon Scientific, Portland, EUA) were used to monitor temperature and relative humidity in the room.

A corn-soybean meal diet was formulated for the initial phase with the following levels: 22.2% crude protein, 2,950 kcal of metabolizable energy/kg of diet, 1.31% digestible lysine, 0.94% digestible methionine + cystine and 0.852% digestible threonine. Animals in the control group (CG) were administered 0.2 mL of Marek’s vaccine suspended in sterile saline solution subcutaneously, whereas the animals in the antimicrobial-administered group (AG) received 0.2 mL of Marek’s vaccine suspended in sterile ceftiofur solution (0.2 mg ceftiofur sodium).

Cloacal swabs were randomly collected from two animals per repetition before vaccination (day 0) and at 3, 5, 7, 9, 11 and 14 days post-hatching. After swab collection the animals were euthanized. The swabs were placed into Luria-Bertani broth (Himedia, India) supplemented with ceftiofur (2mg/L) and incubated at 37°C for 24 h. Then, a 20μL aliquot was spread onto MacConkey Agar (Acumedia, EUA) supplemented with ceftiofur (2mg/L) and incubated at 37°C for 24 h. Lactose fermenting colonies (four per plate) showing characteristics of *E*. *coli* were transferred to Eosin Methylene Blue Agar (EMB) (Himedia, India) and further confirmed as *E*. *coli* by the following biochemical tests: Triple Sugar Iron Agar (TSI) (Himedia, India), Lysine Iron Agar (LIA) (Himedia, India), Sulfide Indole Motility (SIM) (Acumedia, EUA), Simmons Citrate Agar (Oxoid, UK) and Urea Agar Base (Oxoid, UK).

The Clinical Laboratory Standards Institute (CLSI) disk diffusion method [7] was used to test *E*. *coli-* confirmed colonies for antimicrobial susceptibility to the following drugs: amoxicillin/clavulanate (Amx/Clv, 20/10 μg, Cecon, São Paulo, Brazil), aztreonam (ATM, 30 μg, Cecon) cefotaxime (CTX, 30 μg, Cecon), ceftazidime (CAZ, 30 μg, Cecon), ceftriaxone (CRO, 30 μg, Cecon), ciprofloxacin (CIP, 5 μg, Cecon), chloramphenicol (C, 30 μg, Cecon), gentamicin (GM, 10 μg, Cecon), sulfisoxazole / trimethoprim (SXT, 23.75 / 1.25 μg, Cecon) and tetracycline (Te, 30 μg, Cecon).

From each plate, all isolates showing different resistance patterns were taken for further confirmation, therefore, in some cases more than one isolate was recovered per sample (bird). We also determined the minimum inhibitory concentration (MIC) of ceftiofur (CTF) by the broth microdilution method [7] using 96-well microtiter plates containing final ceftiofur concentrations ranging from 0.5 μg/mL to 256 μg/mL. CLSI [7] criteria were used to interpret MIC results as susceptible (MIC ≤2 mg/L), intermediate (4 mg/L), or resistant (≥8 mg/L).

Phenotypic ESBL detection was carried out by double-disk synergy test using CTX, CAZ and CRO disks placed at a distance of 20 mm concentrically to the Amx/Clv disk [7]. *E*. *coli* isolates were also tested by PCR targeting the ESBL genes (*bla*_CTX-M_, *bla*_CTX-M-1_, *bla*_CTX-M-2_, *bla*_CTX-M-8_ and *bla*_SHV_) as well as plasmid-mediated AmpC genes (*bla*_ACC_, *bla*_CMY-2_, *bla*_DHA_, *bla*_FOX_, *bla*_MOX_ and *bla*_MIR_), using primer sequences and conditions as previously described [8–11] and DNA extracted by phenol/chloroform/isoamyl-alcohol (25:24:1) as described by Fritsch et al. [12]. Afterwards, the DNA extracted from confirmed ESBL-producing *E*. *coli* was adjusted to 50 ng/ μL using a microvolume spectrometer (Colibri, Titertek Berthold, Germany) and Enterobacterial Repetitive Intergenic Consensus PCR (ERIC-PCR) was used as genotyping method, as previously described [13]. Shortly, reactions were performed in 25 μL containing 1 pmol of primer, 200 mM of each dNTP, 3 mM of MgCl_2_, 100 ng of genomic DNA, and 1U of Taq DNA polymerase (Invitrogen, Brazil). Amplification was performed in a thermal cycler (TPersonal Thermocycler, Biometra, Germany). Products were analyzed by electrophoresis in 2% agarose gel (LGC Biotechnology, Brazil) stained with GelRed (Biotium, USA). The presence or absence of bands was analyzed visually under ultraviolet light. ERIC-PCR band patterns were scanned and analyzed using the Dice product moment correlation coefficient (2% tolerance) by BioNumerics software (Version 7.1, Applied Maths, Belgium). Clustering analysis was carried out by the unweighted pair group method with arithmetic averages (UPGMA). *E*. *coli* ATCC 25922 was used as internal control (outgroup). Details on the DNA extraction and PCR protocols that were used in this study are described in the Supplementary material (S1 Text and S1 Table). The discriminatory power (D-value) was calculated as described by Hunter [14].

Fisher’s exact test at 5% probability was used to compare the overall frequency of phenotypic ESBL-*E*. *coli* isolates between control group (CG) and antimicrobial-administered group (AG). A Bayesian binomial logistic regression (BLR) approach with 8,000 repetitions was used to infer the probability of the ESBL occurrence between the treatment groups along the experimental period. Statistical analyses were performed in R environment [15] using brms package obtained from CRAN (https://cran.r-project.org/web/packages/brms/index.html)

## Results and discussion

A total of 57 ceftiofur-resistant *E*. *coli* isolates were obtained and confirmed by means of the disk-diffusion method. Out of them, 52 (91.2%) were recovered from the AG group in days 5 (10; 17.5%), 7 (12; 21.1%), 9 (9; 15.8%), 11 (7; 12.3%), and 14 (19; 33.3%). Five (8.8%) ceftiofur-resistant *E*. *coli* were cultured from CG birds; all from the last sampling day (14 days).

According to the antimicrobial susceptibility test, high resistance rates were observed for CTX (100%), Te (100%), CRO (94.7%), STX (54.4%), ATM (42.1%) and CIP (40.4%), corroborating previous reports showing increased antimicrobial resistance rates in bacteria from broilers and layers [1] and pigs [16] treated with third generation cephalosporins.

The lowest resistance rates were seen against CAZ (17.5%), Amx/Clv (14%) and GM (8.8%) (Table 1). All 57 recovered *E*. *coli* isolates were resistant to CTF (MIC ≥ 8 μg/mL), 21% (12/57) of them showing MIC ≥ 128 μg/mL (S3 Table). We observed a perfect agreement between CFT and CTX resistance. This finding supports a previous report comparing MIC values of CFT versus other cephalosporins, including CAZ, in 118 *E*. *coli* from different food-producing animals [17]. Therefore, the use of CTX seems to be a successful approach to assess ceftiofur susceptibility in enterobacteria. That could be of special interest for investigations comparing antimicrobial resistance between isolates from animal and human sources.

**Table 1:** Antimicrobial resistance of 57 *E*. *coli* cultured from chicks receiving ceftiofur added to Marek’s vaccine (AG) and chicks receiving the vaccine only (CG). Isolates were recovered from MacConkey agar supplemented with ceftiofur (2mg / L).

Antimicrobials	Frequency of resistance		
	AG*	CG^#^	Total (%)
Amx/Clv (20 / 10 μg)	8	0	8 (14)
ATM (30 μg)	22	2	24 (42.1)
CTX (30 μg)	52	5	57 (100)
CAZ (30 μg)	10	0	10 (17.5)
CRO (30 μg)	49	5	54 (94.7)
CIP (5 μg)	22	1	23 (40.4)
C (30 μg)	19	1	20 (35.1)
GM (10 μg)	5	0	5 (8.8)
SXT (23.75 / 1.25 μg)	29	2	31 (54.4)
Te (30 μg)	52	5	57 (100)

Antimicrobials tested: Amx/Clv = amoxicillin + clavulanate; ATM = aztreonam; CTX = cefotaxime; CAZ = ceftazidime; CRO = ceftriaxone; CIP = ciprofloxacin; C = chloramphenicol; GM = gentamicin; SXT = sulfisoxazole + trimethoprim; Te = tetracycline.

* antimicrobial-administered group

^#^ control group

Based on the antimicrobial susceptibility test, 63.2% (36/57) of all *E*. *coli* isolates were resistant to three or more different classes of drugs and therefore considered multidrug resistant (MDR) (S2 and S3 Tables). A total of 14 isolates (24.6%) showed resistance to more than five antimicrobial classes.

A total of 24 (42.1%) isolates phenotypically confirmed as ESBL were recovered throughout the experiment and further investigated by PCR (Table 2). Only 3/24 (12.5%) isolates were obtained from CG, whereas 21 (87.5%) were recovered from AG. From the positive isolates, 14 (58.3%) harbored the genes *bla*_CTX-M_ and *bla*_SHV_; 12 out of the 14 originated from AG. Only 2 out of 14 ESBL-*E*. *coli* recovered from CG harbored those genes. *bla*_CTX-M-1_ was the most frequent CTX-M type observed (13/14), whereas only one isolate harbored *bla*_CTXM-8_ (1/14). No *bla*_CTX-M-2_ has been detected among the isolates. Similarly to the findings of our study, *E*. *coli* harboring bla_CTX-M_ genes have been shown to be frequently resistant to CTF and CTX but susceptible to CAZ [18], which is possibly related to the fact that CTX-M β-lactamase cannot hydrolyze CAZ as efficiently as CTX [19].

**Table 2:** Number of animals positive for ESBL-producing *E*. *coli* identified phenotypically by the double-disk synergy test.

Sampling day^a^	Experimental groups						P*
		AG			CG		AG *vs*. CG
	Positive ESBL-*E*. *coli* chicks^b^	MIC^c^	Genes^d^	Positive ESBL-*E*. *coli* chicks	MIC	Genes	
Day 5	5/12	64–256	*bla*_CTX-M-8;_ *bla*_SHV_	0/12	-	-	*
Day 7	5/12	16–256	*bla*_CTX-M-1;_ *bla*_SHV_	0/12	-	-	*
Day 9	2/12	8	*bla*_CTX-M-1;_ *bla*_SHV_	0/12	-	-	*
Day 11	1/12	16	-	0/12	-	-	*
Day 14	8/12	32–64	*bla*_CTX-M-1;_ *bla*_SHV_	3/12	64	*bla*_CTX-M-1;_ *bla*_SHV_	*

^a^ No ESBL-*E*. *coli* recovered at 0 and 3 days

^b^ Frequency of animals harboring ceftiofur-resistant *E*. *coli*

^c^ Range of minimum inhibitory concentration (MIC) of ceftiofur (ug/mL)

^d^ Resistant genes detected by PCR in the isolates

* Significantly different confidence intervals of the predicted probabilities of ESBL *E*. *colli* by Bayesian Binomial Logistic Regression analysis.

The genes *bla*_CTX-M_, *bla*_CTX-M-1_, *bla*_CTX-M-2_, *bla*_CTX-M-8_ and *bla*_SHV_ were not detected in the 33 ceftiofur-resistant *E*. *coli* that were phenotypically negative for the double-disk synergy test for ESBL detection. Furthermore, none of the 57 CFT-resistant *E*. *coli* isolates harbored any of the plasmid-mediated AmpC genes investigated in the present study, including *bla*_CMY-2_, which has been reported in CFT-resistant *E*. *coli* from animal origin in different countries [20–24]. Based on these findings and on the phenotypic results, we assume that the 33 CFT-resistant isolates could harbor other cephamycinase genes that were not investigated in our study or maybe a mutation in the chromosomal *ampC* associated with the hyperproduction of cephamycinase. This last hypothesis is more probable, as cefoxitin-resistance in *E*. *coli* has been less commonly associated to plasmid-mediated AmpC β-lactamases than to hyperproduction of chromosomal AmpC β-lactamase [25].

**Fig 1:**
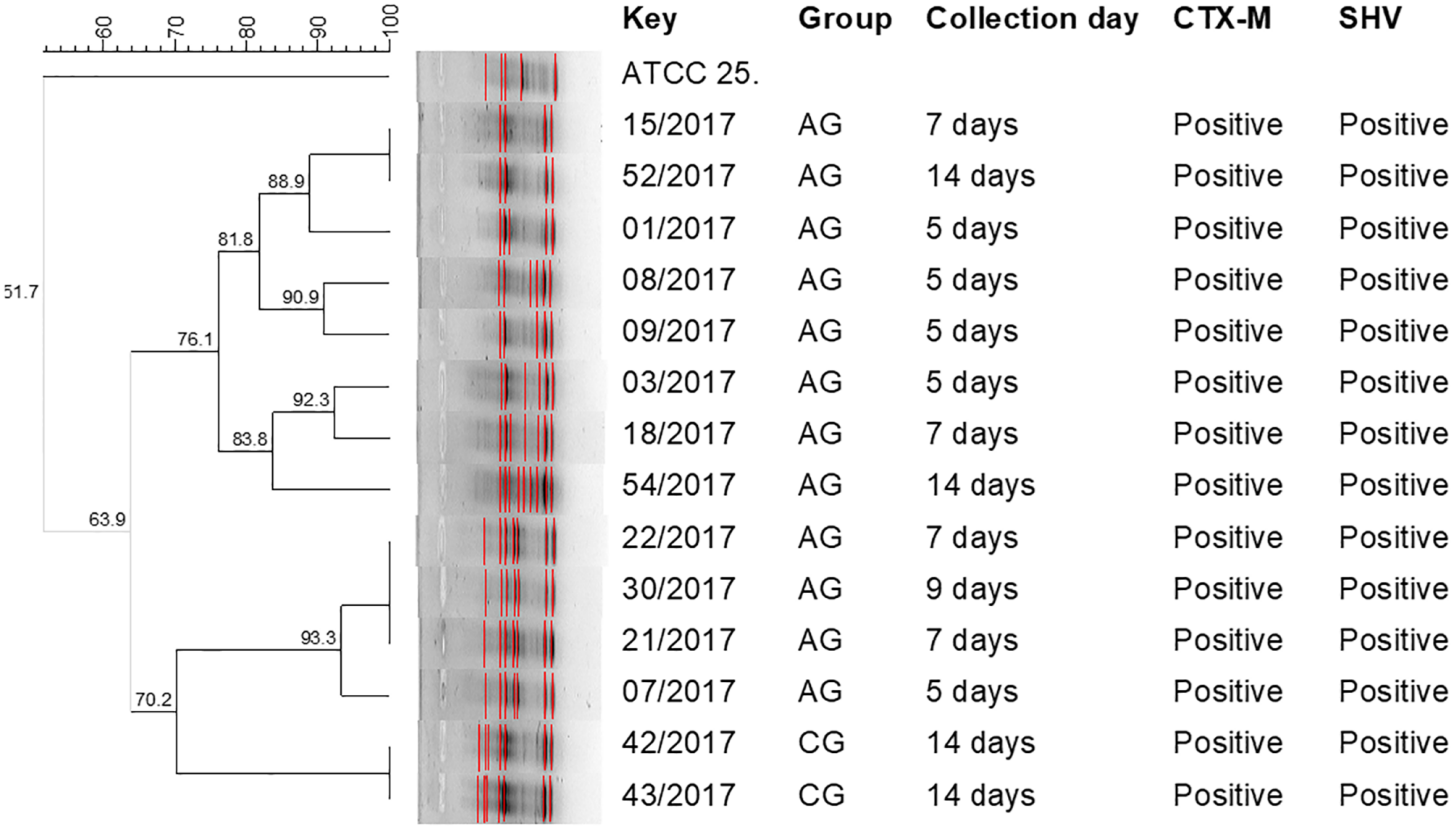
ERIC-PCR genotyping of ESBL-*E*. *coli* cultured from broiler chick cloacal swabs. Dendrogram showing the genotypic similarities among ESBL-positive *E*. *coli* by means of ERIC-PCR. Key = Identification of isolate; AG = antimicrobial-administered group—0.2mL (2 mg/L) of ceftiofur; CG = control group—0.2mL of saline solution.

Interestingly, distinct genotypic patterns among ESBL-producing *E*. *coli* isolates from AG and CG were observed, indicating that isolates of CG animals emerged from a different population. This finding also indicates that there was no cross-contamination of ESBL-*E*. *coli* from AG to CG animals (Fig 1). The ERIC-PCR showed a D-value of 0.96, indicating that the test was highly discriminatory.

Our study demonstrated that the off-label administration of a third-generation cephalosporin is associated (p<0.001) with the presence ESBL-producing *E*. *coli* in the gut of chicks. The Bayesian binomial logistic regression indicated that the probability of ESBL-*E*. *coli* occurrence was significantly higher in treated chicks at 7 and 14 days compared to the probabilities of ESBL-*E*. *coli* across other experimental days, although significant differences were observed between AG and CG groups at 5, 7, 9, 11 and 14 days of age (S1 Fig).

Although the clonal spread of ESBL-*E*. *coli* to humans through the food chain has not been demonstrated yet [26], the most common ESBL found in bacteria from chicken meat and humans, including blood culture specimens, have been shown to be identical [27]. Therefore, foods contaminated by antimicrobial resistant Enterobacteriaceae become reservoirs of ESBL genes [26–30] that can be ultimately acquired by pathogenic bacteria, reducing the efficacy of antimicrobials in veterinary and human medicine. Furthermore, ESBL-producing enterobacteria can disseminate among animals and occupationally exposed workers, as recently demonstrated in swine abattoirs [31].

To our knowledge, this is the first experimental report demonstrating that the common practice of administering ceftiofur to day-one chicks increases the short-term shedding of ESBL-producing *E*. *coli*. The real impact of that on the spread of antimicrobial resistant bacteria to the food chain, final consumers, and environment needs to be further investigated.

## Supporting information

S1 Text DNA extraction by phenol/chloroform/isoamyl-alcohol (25:24:1) protocol. (DOCX)

S1 Table Oligonucleotide primers used to amplify antimicrobial resistance genes (ESBL, AmpC) and enterobacterial repetitive intergenic consensus (ERIC) regions. (DOCX)

S2 Table Antimicrobial resistance profiles of ceftiofur-resistant *E*. *coli* (n = 57) cultured from chicks receiving ceftiofur added to Marek’s vaccine (AG) and chicks receiving the vaccine only (CG). (DOCX)

S3 Table Frequencies of antimicrobial resistance profiles of ceftiofur-resistant *E*. *coli* (n = 57) cultured from chicks receiving ceftiofur added to Marek´s vaccine (AG) and chicks receiving the vaccine only (CG). (DOCX)

S1 Fig Plot showing confidence intervals for the predicted probabilities of ESBL-*E*. *coli* in chicks receiving ceftiofur added to Marek’s vaccine (AG) and chicks receiving the vaccine only (CG). Analysis performed by means of Bayesian binomial logistic regression analysis using 8,000 repetitions in R environment and brms package.
(PDF)
