# Supplementary material for: Off-label use of ceftiofur in one-day chicks triggers a short-term increase of ESBL-producing E. coli in the gut
Source: PLoS One. 2018 Sep 11;13(9):e0203158. doi: 10.1371/journal.pone.0203158 (PMC6133352; doi:10.1371/journal.pone.0203158)
Supplement: S1 Text — (DOCX) [file pone.0203158.s001.docx]

**S1 Text. DNA extraction by phenol/chloroform/isoamyl-alcohol (25:24:1) protocol.**

Briefly, inoculum was grown in BHI broth for 18 h at 37°C and 1.4 mL were then centrifuged at 14,000 rpm/15 min. The supernatant was discarded and the pellet homogenized with 500 μL TE buffer de (1 M Tris- HCl, pH 8,0; 0.5 M EDTA, pH8,0) added of 10 μL lysozyme (10 mg/mL) and Proteinase K (5 mg/mL). This solution was homogenized by vortex and incubated overnight 60°C. Afterwards, 10 μL of STE buffer (1 M Tris- HCl, pH 8,0; 0.5 M EDTA, pH8,0; 5 M NaCl) were added and incubated 15 min at 60°C. 130 μL of ammonium acetate (7.5 M) were added, followed by ice incubation (15 min) and centrifugation (14,000 rpm/15 min). 750 μL of supernatant were transferred to new tube and the same volume of phenol: chloroform: isoamyl alcohol (25:24:1) was added before centrifugation at 14,000 rpm/5 min. In a fresh microtube, 400 μL of supernatant and 420 μL absolute ethanol were added and incubated overnight at -20°C. After centrifugation (14,000 rpm/15 min), the supernatant was discarded, and the pellet resuspended in 30 μL of DNAse/RNAse free water. Concentration and purity were determined at 260/280 and 260/230 using spectrophotometer (Colibri, Titertek Berthold, Germany). All samples were standardized at a concentration of 50 ng/µL before use.
