# Supplementary material for: Off-label use of ceftiofur in one-day chicks triggers a short-term increase of ESBL-producing E. coli in the gut
Source: PLoS One. 2018 Sep 11;13(9):e0203158. doi: 10.1371/journal.pone.0203158 (PMC6133352; doi:10.1371/journal.pone.0203158)
Supplement: S1 Table — (DOCX) [file pone.0203158.s002.docx]

| Gene | Amplicon size  (bp) | Oligonucleotide primer sequences (5’-3’) | Cycling  Conditions* | Reference |
| --- | --- | --- | --- | --- |
| *bla*_SHV_ | 930 | F: GGGTTATTCTTATTTGTCGC  R: TTAGCGTTGCCAGTGCTC | 1 | 8 |
| *bla*_CTX-M_ | 554 | F: SCSATGTGCAGYACCAGTAA  R: CCGCRAT ATGRTTGGTGGTG | 2 | 9 |
| *bla*_CTX-M_*_-1_* | 854 | F: AAATCACTGCGYCAGTTCA  R: GGTGACGATTTTAGCCGCCG | 3 | 10 |
| *bla*_CTX-M_*_-2_* | 870 | F: GACTCAGAGCATTCGCCGC  R: TCAGAAACCGYGGGTTACGA | 3 | 10 |
| *bla*_CTX-M_*_-8_* | 861 | F: GATGAGACATCGCGTTAAG  R: GGTGACGATTTTCGCGGCA | 3 | 10 |
| *bla*_MOX_ | 520 | F: GATCGGATTGGAGAACCAGA  R: ATTTCTGACCGCATTTCCAT | 4 | 11 |
| *bla*_CMY-2_ | 462 | F: TGGCCAGAACTGACAGGCAAA  R: TTTCTCCTGAACGTGGCTGGC | 4 | 11 |
| *bla*_DHA_ | 405 | F: AACTTTCACAGGTGTGCTGGGT  R: CCGTACGCATACTGGCTTTGC | 4 | 11 |
| *bla*_ACC_ | 346 | F: AACAGCCTCAGCAGCCGGTTA  R: TTCGCCGCAATCATCCCTAGC | 4 | 11 |
| *bla*_MIR_ | 302 | F: TCGGTAAAGCCGATGTTGCGG  R: CTTCCACTGCGGCTGCCAGTT | 4 | 11 |
| *bla*_FOX_ | 190 | F: AACATGGGGTATCAGGGAGATG  R: CAAAGCGCGTAACCGGATTGG | 4 | 11 |
| ERIC | Variable | 1R: ATGTAAGCTCCTGGGGATTCAC | 5 | 13 |

**S1 Table. Oligonucleotide primers used to amplify the antimicrobial resistance genes (ESBL, AmpC) and enterobacterial repetitive intergeneric consensus (ERIC) region**

Condition 1 = 94°C 5 min, and 30 cycles at 94ºC 1 min, 54°C 1 min, 72°C 1 min; and 72ºC 5 min. Condition 2 = 94ºC 3 min, 35 cycles at 94ºC 30 sec, 55º 30 sec, 72ºC 45 sec for 35 cycles, and 72°C 5 min. Condition 3 = 94ºC 5 min, 30 cycles at 94ºC 45 sec, 60ºC 1 min, 72ºC 1 min; and 72ºC 10 min. Condition 4 = 94°C 3 min, 25 cycles at 94°C 30s, 64°C for 30s, and 72°C 1 min; and 72°C 7 min. Condition 5 = 94ºC 3 min, 30 cycles at 94ºC 1 min, 50º 1 min, 72ºC 2 min; and 72°C 5 min.
