# Supplementary material for: Off-label use of ceftiofur in one-day chicks triggers a short-term increase of ESBL-producing E. coli in the gut
Source: PLoS One. 2018 Sep 11;13(9):e0203158. doi: 10.1371/journal.pone.0203158 (PMC6133352; doi:10.1371/journal.pone.0203158)
Supplement: S2 Table — (DOCX) [file pone.0203158.s003.docx]

**S2 Table. Antimicrobial resistance profiles ceftiofur-resistant *E. coli* (n=57) cultured from chicks receiving ceftiofur added to Marek´s vaccine (AG) and chicks receiving the vaccine only (CG).**

| **Isolate ID** | **Group** | **Repetition** | **Sampling day** | **Resistance profile^a^** | **MIC ceftiofur µg/ml** | **ESBL double-disk synergy test** | ***bla*_CTX-M_** | ***bla*_CTX-M-1_** | ***bla*_CTX-M-2_** | ***bla*_CTX- M- 8_** | ***bla*_SHV_** | **MDR^b^** |
| --- | --- | --- | --- | --- | --- | --- | --- | --- | --- | --- | --- | --- |
| 1/2017 | AG | R1 | Day 5 | CTX, Te, C, SXT, CIP | 64 | + | + | - | - | + | + | + |
| 2/2017 | AG | R1 | Day 5 | CRO, CTX, Te, C, SXT, CIP | 128 | - | - | - | - | - | - | + |
| 3/2017 | AG | R3 | Day 5 | CRO, CTX, GM, Te | 256 | + | + | + | - | - | + | + |
| 4/2017 | AG | R3 | Day 5 | CRO, CTX, ATM, Te | 256 | - | - | - | - | - | - | - |
| 5/2017 | AG | R3 | Day 5 | CRO, CTX, ATM, Te, SXT | 256 | - | - | - | - | - | - | + |
| 6/2017 | AG | R3 | Day 5 | CRO, CTX, ATM, CAZ, Amx/Clv, GM, Te | 256 | - | - | - | - | - | - | + |
| 7/2017 | AG | R4 | Day 5 | CRO, CTX, Te | 256 | + | + | + | - | - | + | - |
| 8/2017 | AG | R5 | Day 5 | CRO, CTX, Te, CIP | 256 | + | + | + | - | - | + | + |
| 9/2017 | AG | R6 | Day 5 | CRO, CTX, GM, Te | 64 | + | + | + | - | - | + | + |
| 10/2017 | AG | R6 | Day 5 | CRO, CTX, ATM, CAZ, Amx/Clv, Te, SXT | 128 | - | - | - | - | - | - | + |
| 11/2017 | AG | R1 | Day 7 | CRO, CTX, ATM, Amx/Clv, Te, C, SXT, CIP | 8 | - | - | - | - | - | - | + |
| 12/2017 | AG | R1 | Day 7 | CRO, CTX, Amx/Clv, Te, C, SXT, CIP | 16 | - | - | - | - | - | - | + |
| 13/2017 | AG | R1 | Day 7 | CRO, CTX, Amx/Clv, Te, C, SXT, CIP | 64 | - | - | - | - | - | - | + |
| 14/2017 | AG | R1 | Day 7 | CRO, CTX, Te, C, SXT, CIP | 32 | - | - | - | - | - | - | + |
| 15/2017 | AG | R1 | Day 7 | CRO, CTX, ATM, CAZ, Te, SXT | 16 | + | + | + | - | - | + | + |
| 16/2017 | AG | R1 | Day 7 | CRO, CTX, ATM, CAZ, Te, SXT | 16 | - | - | - | - | - | - | + |
| 17/2017 | AG | R3 | Day 7 | CRO, CTX, ATM, CAZ, Te | 16 | - | - | - | - | - | - | - |
| 18/2017 | AG | R3 | Day 7 | CRO, CTX, ATM, Te | 32 | + | + | + | - | - | + | - |
| 19/2017 | AG | R3 | Day 7 | CRO, CTX, ATM, Te | 16 | - | - | - | - | - | - | - |
| 20/2017 | AG | R4 | Day 7 | CRO, CTX, Te | 16 | + | - | - | - | - | - | - |
| 21/2017 | AG | R5 | Day 7 | CRO, CTX, ATM, Te | 16 | + | + | + | - | - | + | - |
| 22/2017 | AG | R5 | Day 7 | CRO, CTX, ATM, Te | 256 | + | + | + | - | - | + | - |
| 23/2017 | AG | R1 | Day 9 | CRO, CTX, Te, C, SXT, CIP | 64 | + | - | - | - | - | - | + |
| 24/2017 | AG | R3 | Day 9 | CRO, CTX, ATM, Te | 8 | - | - | - | - | - | - | - |
| 25/2017 | AG | R3 | Day 9 | CRO, CTX, ATM, CAZ, Te | 32 | - | - | - | - | - | - | - |
| 26/2017 | AG | R3 | Day 9 | CRO, CTX, ATM, Te | 16 | - | - | - | - | - | - | - |
| 27/2017 | AG | R3 | Day 9 | CRO, CTX, Te | 8 | - | - | - | - | - | - | - |
| 28/2017 | AG | R4 | Day 9 | CRO, CTX, ATM, Te, C | 8 | - | - | - | - | - | - | + |
| 29/2017 | AG | R4 | Day 9 | CRO, CTX, ATM, CAZ, Te, SXT | 16 | - | - | - | - | - | - | + |
| 30/2017 | AG | R4 | Day 9 | CRO, CTX, Te | 8 | + | + | + | - | - | + | - |
| 31/2017 | AG | R4 | Day 9 | CRO, CTX, ATM, CAZ, Amx/Clv, GM, Te, SXT | 8 | - | - | - | - | - | - | + |
| 32/2017 | AG | R1 | Day 11 | CRO, CTX, Te, C, SXT, CIP | 16 | + | - | - | - | - | - | + |
| 33/2017 | AG | R1 | Day 11 | CRO, CTX, Te, C, SXT, CIP | 8 | - | - | - | - | - | - | + |
| 34/2017 | AG | R1 | Day 11 | CRO, CTX, GM, Te, SXT, CIP | 8 | - | - | - | - | - | - | + |
| 35/2017 | AG | R1 | Day 11 | CRO, CTX, Amx/Clv, Te, C, SXT, CIP | 8 | - | - | - | - | - | - | + |
| 36/2017 | AG | R3 | Day 11 | CRO, CTX, ATM, CAZ, Te, SXT | 32 | - | - | - | - | - | - | + |
| 37/2017 | AG | R6 | Day 11 | CRO, CTX, ATM, Te, SXT | 16 | - | - | - | - | - | - | + |
| 38/2017 | AG | R6 | Day 11 | CRO, CTX, ATM, CAZ, Amx/Clv, Te | 32 | - | - | - | - | - | - | - |
| 39/2017 | CG | R1 | Day 14 | CRO, CTX, Te, C, SXT, CIP | 16 | + | - | - | - | - | - | + |
| 40/2017 | CG | R3 | Day 14 | CRO, CTX, ATM, Te | 32 | - | - | - | - | - | - | - |
| 41/2017 | CG | R3 | Day 14 | CRO, CTX, Te, SXT | 128 | - | - | - | - | - | - | + |
| 42/2017 | CG | R3 | Day 14 | CRO, CTX, Te | 64 | + | + | + | - | - | + | - |
| 43/2017 | CG | R3 | Day 14 | CRO, CTX, ATM, Te | 64 | + | + | + | - | - | + | - |
| 44/2017 | AG | R1 | Day 14 | CRO, CTX, Te, C, SXT, CIP | 64 | + | - | - | - | - | - | + |
| 45/2017 | AG | R1 | Day 14 | CRO, CTX, Te, C, SXT, CIP | 64 | + | - | - | - | - | - | + |
| 46/2017 | AG | R1 | Day 14 | CRO, CTX, Te, C, SXT, CIP | 128 | - | - | - | - | - | - | + |
| 47/2017 | AG | R1 | Day 14 | CRO, CTX, Te, C, SXT, CIP | 64 | - | - | - | - | - | - | + |
| 48/2017 | AG | R2 | Day 14 | CRO, CTX, Te, C, SXT, CIP | 8 | + | - | - | - | - | - | + |
| 49/2017 | AG | R2 | Day 14 | CTX, Te, C, SXT, CIP | 128 | + | - | - | - | - | - | + |
| 50/2017 | AG | R2 | Day 14 | CTX, Te, C, SXT, CIP | 8 | + | - | - | - | - | - | - |
| 51/2017 | AG | R3 | Day 14 | CRO, CTX, ATM, Te | 32 | - | - | - | - | - | - | - |
| 52/2017 | AG | R3 | Day 14 | CRO, CTX, Te | 32 | + | + | + | - | - | + | - |
| 53/2017 | AG | R3 | Day 14 | CRO, CTX, Te | 64 | - | - | - | - | - | - | - |
| 54/2017 | AG | R4 | Day 14 | CRO, CTX, Te | 64 | + | + | + | - | - | + | - |
| 55/2017 | AG | R4 | Day 14 | CRO, CTX, Te, C, SXT, CIP | 64 | + | - | - | - | - | - | + |
| 56/2017 | AG | R4 | Day 14 | CRO, CTX, Te, C, SXT, CIP | 16 | - | - | - | - | - | - | + |
| 57/2017 | AG | R4 | Day 14 | CRO, CTX, Te, C, SXT, CIP | 64 | - | - | - | - | - | - | + |

1. Amx/Clv, amoxicillin/clavulanate; ATM, aztreonam; CTX, cefotaxime; CAZ, ceftazidime; CRO, ceftriaxone; CIP, ciprofloxacin; C, chloramphenicol; GM, gentamicina; SXT, sulfisoxazole/trimethoprim; Te, tetracycline.
2. MDR = multidrug resistant isolate (resistance to three or more different classes of drugs)

*All 57 *E. coli* isolates tested negative for AmpC genes (*bla*_ACC_, *bla*_CMY-2_, *bla*_DHA_, *bla*_FOX_, *bla*_MOX_ and *bla*_MIR_).
