# Supplementary material for: Off-label use of ceftiofur in one-day chicks triggers a short-term increase of ESBL-producing E. coli in the gut
Source: PLoS One. 2018 Sep 11;13(9):e0203158. doi: 10.1371/journal.pone.0203158 (PMC6133352; doi:10.1371/journal.pone.0203158)
Supplement: S3 Table — (DOCX) [file pone.0203158.s004.docx]

**S3 Table. Frequencies of antimicrobial resistance profiles of ceftiofur-resistant *E. coli* (n=57) cultured from chicks receiving ceftiofur added to Marek´s vaccine (AG) and chicks receiving the vaccine only (CG).**

| Group | Resistance Pattern | Frequency (%) |
| --- | --- | --- |
| AG | CROCTXTeCSXTCIP | 13 (22.8%) |
| AG | CROCTXATMTe | 08 (14.03%) |
| AG | CROCTXTe | 07 (12.3%) |
| AG | CROCTXATMCAZTeSXT | 04 (7.01%) |
| AG | CTXTeCSXTCIP | 03 (5.3%) |
| AG | CROCTXAmxTeCSXTCIP | 03 (5.3%) |
| AG | CROCTXATMTeSXT | 02 (3.5%) |
| AG | CROCTXATMCAZTe | 02 (3.5%) |
| AG | CROCTXGMTe | 01 (1.7%) |
| AG | CROCTXATNCAZAmxGMTe | 01 (1.7%) |
| AG | CROCTXTeCIP | 01 (1.7%) |
| AG | CROCTXGMTe | 01 (1.7%) |
| AG | CROCTXATMCAZAmxTeSXT | 01 (1.7%) |
| AG | CROCTXATMAmxTeCSXTCIP | 01 (1.7%) |
| AG | CROCTXATMTeC | 01 (1.7%) |
| AG | CROCTXATMCAZAmxGMTeSXT | 01 (1.7%) |
| AG | CROCTXGMTeSXTCIP | 01 (1.7%) |
| AG | CROCTXATMCAZAmxTe | 01 (1.7%) |
| CG | CROCTXATMTe | 02 (3.5%) |
| CG | CROCTXTeCSXTCIP | 01 (1.7%) |
| CG | CROCTXTeSXT | 01 (1.7%) |
| CG | CROCTXTe | 01 (1.7%) |

Amx/Clv, amoxicillin/clavulanate; ATM, aztreonam; CTX, cefotaxime; CAZ, ceftazidime; CRO, ceftriaxone; CIP, ciprofloxacin; C, chloramphenicol; GM, gentamicina; SXT, sulfisoxazole/trimethoprim; Te, tetracycline. 2015 CLSI breakpoints were used to interpret disk diffusion results.
