# Supplementary figures and images for: Off-label use of ceftiofur in one-day chicks triggers a short-term increase of ESBL-producing E. coli in the gut
Source: PLoS One. 2018 Sep 11;13(9):e0203158. doi: 10.1371/journal.pone.0203158 (PMC6133352; doi:10.1371/journal.pone.0203158)

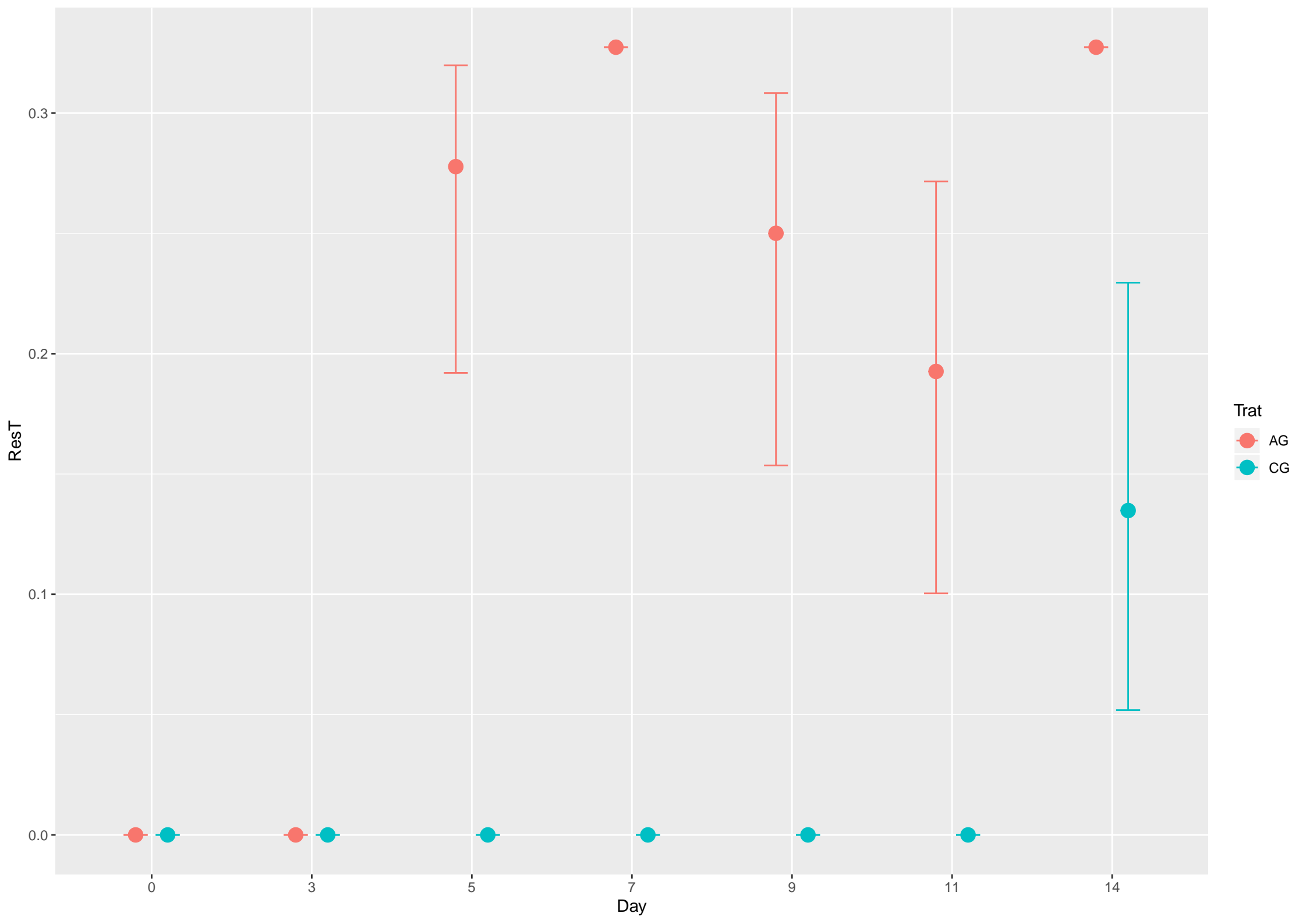

Supplement: S1 Fig — Analysis performed by means of Bayesian binomial logistic regression analysis using 8,000 repetitions in R environment and brms package. (PDF) [file pone.0203158.s005.pdf]
